# The Impact of Serum Levels of Vitamin D3 and Its Metabolites on the Prognosis and Disease Severity of COVID-19

**DOI:** 10.3390/nu14245329

**Published:** 2022-12-15

**Authors:** Hani M. J. Khojah, Sameh A. Ahmed, Sultan S. Al-Thagfan, Yaser M. Alahmadi, Yasser A. Abdou

**Affiliations:** 1 Department of Clinical and Hospital Pharmacy, College of Pharmacy, Taibah University, Al Madinah Al Munawarah 30001, Saudi Arabia; 2 Department of Pharmacognosy and Pharmaceutical Chemistry, College of Pharmacy, Taibah University, Al Madinah Al Munawarah 30001, Saudi Arabia; 3 Ohud Hospital, Al Madinah Al Munawarah 42354, Saudi Arabia

**Keywords:** COVID-19, vitamin D, vitamin D3, cholecalciferol, calcifediol, calcitriol, angiotensin-converting enzyme 2, interleukin-6, NLR

## Abstract

Vitamin D is among the increasingly consumed dietary supplements during the COVID-19 pandemic. It plays a regulatory role in the immune system and moderates the renin–angiotensin system, which is implicated in infection pathogenesis. However, the investigation of serum levels of vitamin D3 forms and their relative ratios in COVID-19 patients is worth investigation to understand the impacts of disease severity. Hence, we investigated the serum levels of vitamin D3 (cholecalciferol) and its metabolites (calcifediol and calcitriol), in addition to their relative ratios and correlations with angiotensin-converting enzyme 2 (ACE2), interleukin-6 (Il-6), and neutrophil–lymphocyte ratio (NLR) in COVID-19 patients compared with healthy controls. Oropharyngeal specimens were collected from the study subjects for polymerase chain reaction testing for COVID-19. Whole blood samples were obtained for blood count and NLR testing, and sera were used for the analysis of the levels of the vitamin and its metabolites, ACE2, and IL-6. We enrolled 103 patients and 50 controls. ACE2, Il-6, and NLR were significantly higher in the patients group (72.37 ± 18.67 vs. 32.36 ± 11.27 U/L, 95.84 ± 25.23 vs. 2.76 ± 0.62 pg/mL, and 1.61 ± 0.30 vs. 1.07 ± 0.16, respectively). Cholecalciferol, calcifediol, and calcitriol were significantly lower in patients (18.50 ± 5.36 vs. 29.13 ± 4.94 ng/mL, 14.60 ± 3.30 vs. 23.10 ± 3.02 ng/mL, and 42.90 ± 8.44 vs. 65.15 ± 7.11 pg/mL, respectively). However, their relative ratios were normal in both groups. Levels of the vitamin and metabolites were strongly positively, strongly negatively, and moderately negatively correlated with ACE2, Il-6, and NLR, respectively. COVID-19 infection severity is associated with a significant decrease in vitamin D3 and its metabolites in a parallel pattern, and with a significant increase in ACE2, Il-6, and NLR. Higher levels of vitamin D and its metabolites are potentially protective against severe infection.

## 1. Introduction

The COVID-19 outbreak has triggered a global public health crisis [1]. In addition, little is known about the preventive factors for this infection [2,3]. Therefore, preventive health measures that reduce the risk of infection, progression, and severity are highly recommended [4]. The global consumption of dietary supplements, including vitamins, zinc, and several natural products, has increased since the beginning of the pandemic [5]. Vitamin D has been reported to have a regulatory impact on the immune system, as a number of immune-based disorders are associated with vitamin D deficiency [6,7,8]. Moreover, vitamin D was found to play a moderating role in the renin–angiotensin–aldosterone system (RAAS) involved in the pathogenesis of COVID-19 [9].

The SARS-CoV-2 virus can identify human and some animal cells, in which the angiotensin-converting enzyme 2 (ACE2) acts as a functional receptor for the virus [10]. This zinc-metallopeptidase is expressed in several organs such as the heart, gastrointestinal tract, and kidneys, but its high expression in the alveolar epithelium of the lungs is directly linked with the human-to-human and cross-species propagation of SARS-CoV-2 [11,12,13]. ACE2 counteracts the action of ACE in such a way that it promotes the conversion of the vasoconstrictor angiotensin II into the vasodilator angiotensin 1–7, which opposes the action of angiotensin II by stimulating the synthesis of nitric oxide [14]. The binding of the virus to ACE2 decreases the expression of the latter, leading to excessive production of angiotensin II (by ACE), which enhances the formation of free radicals and the proinflammatory cytokine, and hence causes lung injury and pneumonia [9].

Vitamin D was found to inhibit renin production and consequently ACE, angiotensin II, and cytokine, and to enhance ACE2 and angiotensin [1,2,3,4,5,6,7]. This is supposed to protect against acute lung injury and acute respiratory distress syndrome, thus ameliorating the respiratory effects of COVID-19 infection [15,16,17]. Vitamin D exists as a group of fat-soluble secosteroids, of which vitamin D3 (cholecalciferol) and vitamin D2 (ergocalciferol) are the most important variants in humans [18]. Cholecalciferol is metabolized in the liver to calcifediol (25-hydroxycholecalciferol), which is further hydroxylated by the kidneys and liver to calcitriol (1,25-dihydroxycholecalciferol), the active form of the vitamin D [19]. Levels of the latter three forms of the vitamin are currently used as biomarkers for vitamin D status, and their decrease indicates vitamin D deficiency in various disorders such as osteoporosis, hyperparathyroidism, certain autoimmune disorders, and vitamin deficiency states [20].

Serum levels of calcitriol are primarily controlled by enzymes of the cytochrome P450 family. Of these, the 25-hydroxylase (CYP27A1) activity is substrate-dependent and is stimulated by the availability of cholecalciferol but not inhibited by calcifediol concentrations [21]. In contrast, the variable activity of 1-α-hydroxylase (CYP27B1) is substrate-independent and is controlled by several hormones and chemicals, including calcitriol, which induce concentration-dependent negative feedback [22]. Vitamin D levels have been assessed in previous studies using either serum levels of calcifediol or levels of calcitriol. The synthesis route of these metabolites suggests that efficient management of vitamin D hydroxylation may be significant in the prediction of its role in COVID-19 disease treatment [9]. Vitamin D and its metabolites usually exist in relative levels to each other. The vitamin D metabolite ratio (VMR) has been proposed as a better predictor of vitamin D status. VMR was measured in type 2 diabetes patients who were at high risk of vitamin D deficiency and was linked to diabetic complications [23]. In addition, calcitriol/calcifediol ratio was reported as a marker of vitamin D hydroxylation efficiency with an average normal ratio of 2.22 (1.64–3.00 pg/ng) [24]. In contrast to individual assessments of the vitamin and its metabolites, VMR can more accurately represent the effectiveness of vitamin D hydroxylases, which can help with substitutive therapeutic therapy for various individuals [24].

Several studies have suggested a strong association between levels of vitamin D metabolites and the disease severity and/or mortality associated with COVID-19 [25,26]. The risk of COVID-19 infection was found to increase with low vitamin D serum level [27,28]. In addition, the deficiency of the vitamin was linked with poor prognosis of the infection [28]. A recent study reported that vitamin D can directly inhibit viral replication while simultaneously acting as an anti-inflammatory and immunomodulatory agent [29]. Additionally, COVID-19 appears to predominantly take advantage of immune evasion during infection, which is a documented pathogenic mechanism for the development of acute respiratory distress syndrome (ARDS), which is followed in some individuals by hyper-response and cytokine storm [30]. The SARS-CoV-2 virus enters alveolar and intestinal epithelial cells via ACE2 as the host receptor. Following deregulation of the renin–angiotensin system, increased cytokine production may result in potentially deadly ARDS [31]. Moreover, the risk of viral infection and death are decreased by vitamin D through several mechanisms. These include preserving cell and gap junctions, boosting cellular immunity by lowering the cytokine storm with effects on interferon γ and tumor necrosis factor alpha (TNF-α) [32], and control of adaptive immunity by suppression of T helper cell type 1 responses and activation of T cells [33].

**Figure 1:**
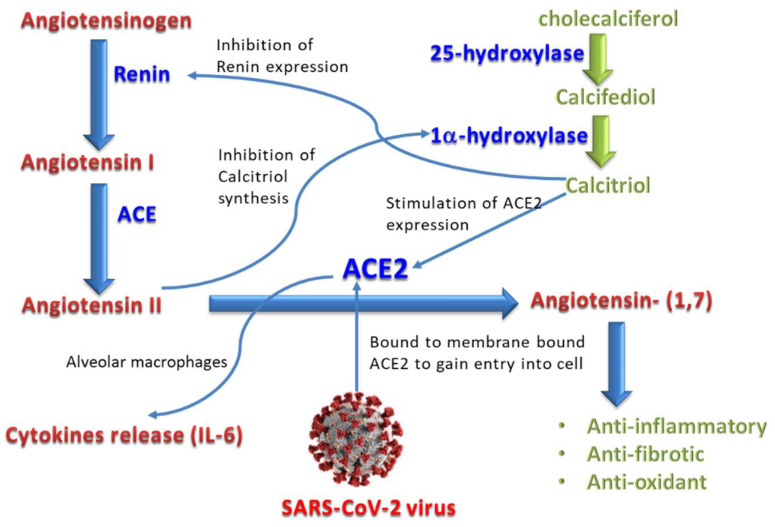
The interaction of Vitamin D3 (cholecalciferol) and its metabolites with the renin–angiotensin System (RAS) and IL-6 in COVID-19 disease.

Evidence suggests that the level of interleukin-6 (IL-6) is more accurate than the C-reactive protein (CRP) and other inflammatory indicators in expecting respiratory failure in COVID-19. Immune dysregulation and ARDS in COVID-19 appear to be most significantly influenced by IL-6 [34]. IL-6 secretion in the lung is increased by the proinflammatory cytokines, particularly interleukin-1β (IL-1β) and TNF-α. An important factor in the induction and propagation of the cytokine storm that causes ARDS and lung injury is the overexpression of IL-6 [35]. It is thought that IL-6 increases lung capillary permeability, which has a significant impact on ARDS development, and promotes the coagulation pathway, which results in microthrombi in the circulation of the lungs and raises the chance for thrombotic event [36]. The interaction of Vitamin D3 and its metabolites with the renin–angiotensin System (RAS) and IL-6 in COVID-19 is presented in Figure 1.

The neutrophil-to-lymphocyte ratio (NLR) is a valuable measure of systemic inflammation and is being evaluated as a guide for the prognosis of many illnesses, with patients with high NLR being actively followed and controlled [25]. Recently, NLR has been identified as a useful tool in identifying COVID-19 infection [37]. In COVID-19 patients, a relationship between elevated NLR and illness severity has also been demonstrated. Although its usage alone in COVID-19 diagnosis may not be particularly accurate, it should be quite effective in ruling out the infection when combined with a comprehensive physical examination [25]. Additionally, it was revealed that there was a substantial relationship between low serum vitamin D levels and NLR in COVID-19 patients [38].

This study aimed to investigate the levels of cholecalciferol and its main metabolites, calcifediol and calcitriol, in the sera of COVID-19 patients compared with healthy individuals. As far as we know, this is the first study that reports comparative serum levels of all forms of vitamin D3 and their relative ratios in COVID-19 patients. In addition, the ratio of these levels and the correlations between them and the levels of ACE2, Il-6, and NLR were investigated. Finally, the possible effect of COVID-19 infection on cholecalciferol metabolism and activation was assessed.

## 2. Materials and Methods

This cross-sectional investigation was carried out between October 2020 and December 2021. Study subjects were enrolled at Ohud Hospital, Madinah, Saudi Arabia, where the COVID-19 patients included were followed up and given the treatment according to the protocol approved by the Saudi Ministry of Health.

### 2.1. Selection of Study Subjects

Patients were chosen based on positive polymerase chain reaction (PCR) testing for COVID-19 as well as the presence of respiratory symptoms such as cough, shortness of breath, and/or lung computed tomography (CT) scans exhibiting ground glass opacity. Controls were selected based on negative PCR and symptoms. Only subjects who agreed to participate in the study were included. The study excluded patients who had comorbid conditions such liver illness, haematological disorders, chronic lung diseases, and those who received radiation and chemotherapy.

### 2.2. Sample Collection and Handling

Oropharyngeal specimens for PCR testing were collected by trained professionals from the Infection Control Unit under restricted conditions for COVID-19. Virus transport medium was used to convey specimens to the laboratory. On the other hand, blood samples were collected under aseptic safety measures by venipuncture. Parts of the samples were centrifuged on-site at 2500× *g* for 10 min at 4 °C. The sera were removed from the samples and maintained at −30 °C until the time of the vitamin D3 assay, in addition to the levels of ACE2, IL-6, and glucose. The remaining parts of the whole blood samples were analyzed for complete blood count (CBC).

### 2.3. Specimen Testing for COVID-19

The oropharyngeal specimens were subjected to viral ribonucleic acid (RNA) extraction followed by complementary deoxyribonucleic acid (cDNA) synthesis. Real-time quantitative PCR (RT-qPCR) was used to test for SARS-CoV-2 using particular probes and primers in accordance with the kit’s instructions. Finally, samples were classified as either positive or negative for COVID-19 based on the PCR findings.

### 2.4. Blood Sample Analyses

Serum levels of vitamin D3 (cholecalciferol) and its metabolites (calcifediol and calcitriol) were quantified by a novel method using an ultra performance liquid chromatography (UPLC) system with a tandem mass detector system (Waters Quattro Premier XE mass spectrometer, Waters Corporation (Milford, MA, USA) [39]. Detection limits were 50 ng/mL for cholecalciferol, 20 ng/mL for calcifediol, and 20 pg/mL for calcitriol. The level of ACE2 was measured in the sera using a sandwich Enzyme-Linked Immunosorbent Assay (ELISA) (ACE2 Kit, Invitrogen, from (ThermoFisher Scientific, Indianapolis, IN, USA)). In addition, serum level of IL-6 was determined by ELISA (ab46027, Abcam plc, Cambridge, UK) according to the manufacturer’s protocols.

### 2.5. Statistical Analysis

The Statistical Package for the Social Sciences, 20th version, from International Business Machines Corp. (Armonk, NY, USA), was employed. Chi-square test, students’ unpaired *t*-tests, and analysis of variance were used to examine the data. Results were presented as mean values ± standard error. Significance was assessed by the *p*-value, where *p* ≤ 0.05 and *p* ≤ 0.001 indicated significant and highly significant differences, respectively.

## 3. Results

This study included 103 COVID-19 patients and 50 healthy controls. According to Table 1, there were no significant variations in the demographic characteristics of the two groups. Shadows or ground-glass opacities were visible in the CT images of suspected patients. They did, however, go through a confirming diagnostic process that included nucleic acid analysis testing and the collection of oropharyngeal swab samples. As depicted in Table 2, COVID-19 patients had substantially greater levels of ACE2, IL-6, glucose, total white blood cells (WBCs), neutrophils, NLR, platelets, mean corpuscular hemoglobin (MCH), and mean corpuscular hemoglobin concentration (MCHC) than healthy participants. In contrast, lymphocytes, red blood cells (RBCs), hemoglobin, and hematocrit were significantly to highly significantly lower in the patients group. However, the difference was nonsignificant for eosinophils and mean corpuscular volume (MCV).

Serum levels of vitamin D3 and its metabolites were highly significantly lower in the COVID-19 patients group compared with the healthy subjects, as demonstrated in Table 3. The calcitriol/calcifediol ratio (VMR1) was 2.97 ± 0.30 pg/ng in COVID-19 patients compared to 2.86 ± 0.46 in healthy subjects, which was within the normal ratio range that was reported to be 1.64–3.00 pg/ng (mean = 2.22) [24]. Similarly, VMR2 (calcifediol/cholecalciferol ratio) was found to be normal (0.82 ± 0.14 ng/ng) in COVID-19 patients compared to 0.81 ± 0.17 in healthy subjects, where the normal VMR2 was reported as 0.8 ± 0.15 ng/ng [40]. The differences between the COVID-19 patients group and healthy group were nonsignificant for both VMR1 and VMR2.

**Figure 2:**
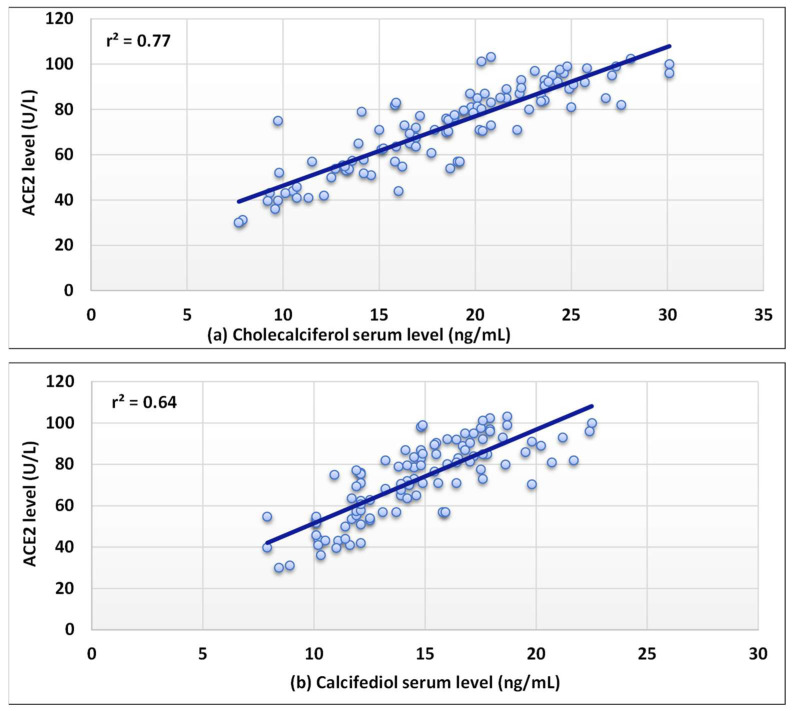

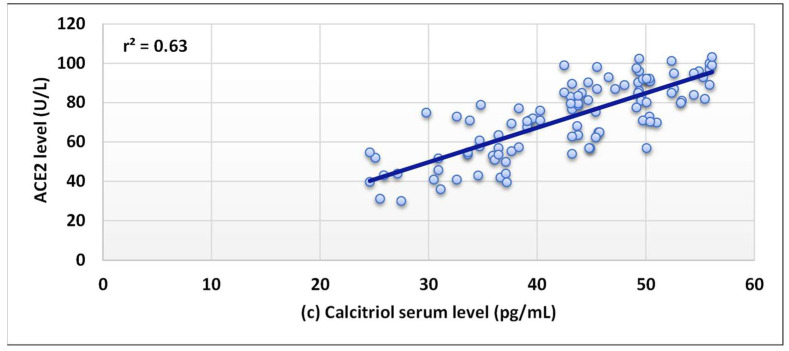
Correlation between the levels of angiotensin-converting enzyme 2 (ACE2) and (**a**–**c**) in COVID-19 patients.

**Figure 3:**
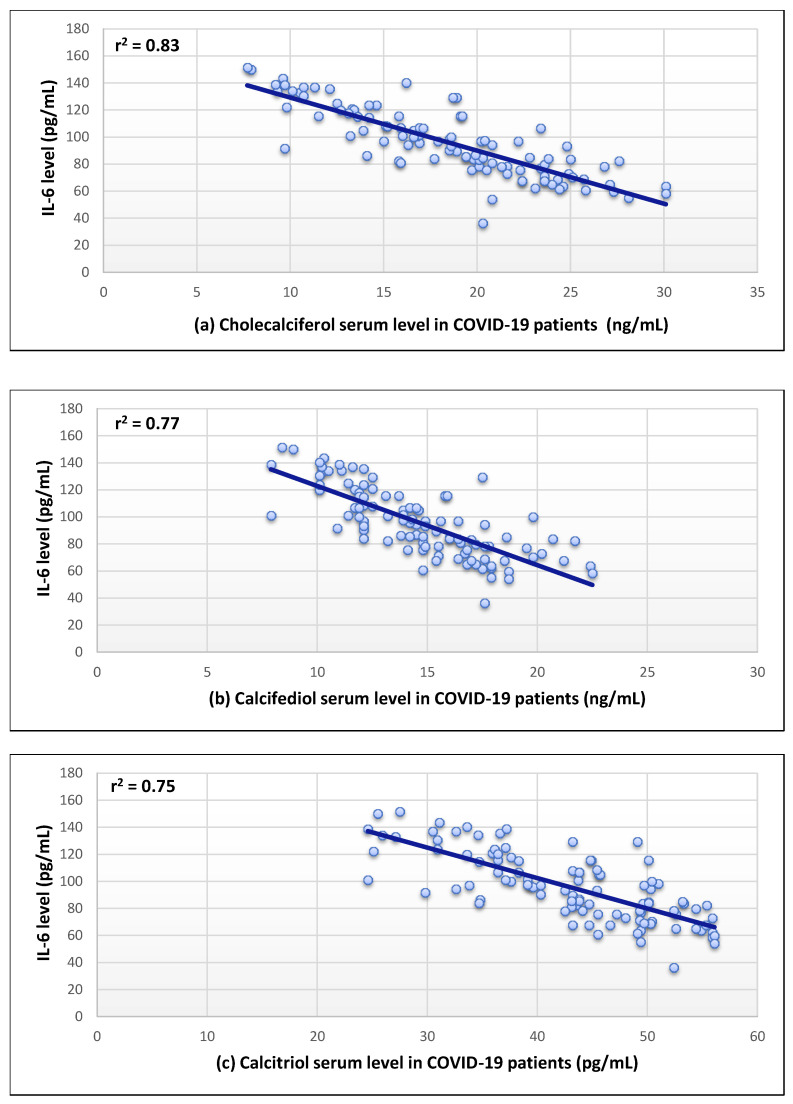
Correlation between the levels of interleukin-6 (IL-6) and (**a**–**c**) in COVID-19 patients.

**Figure 4:**
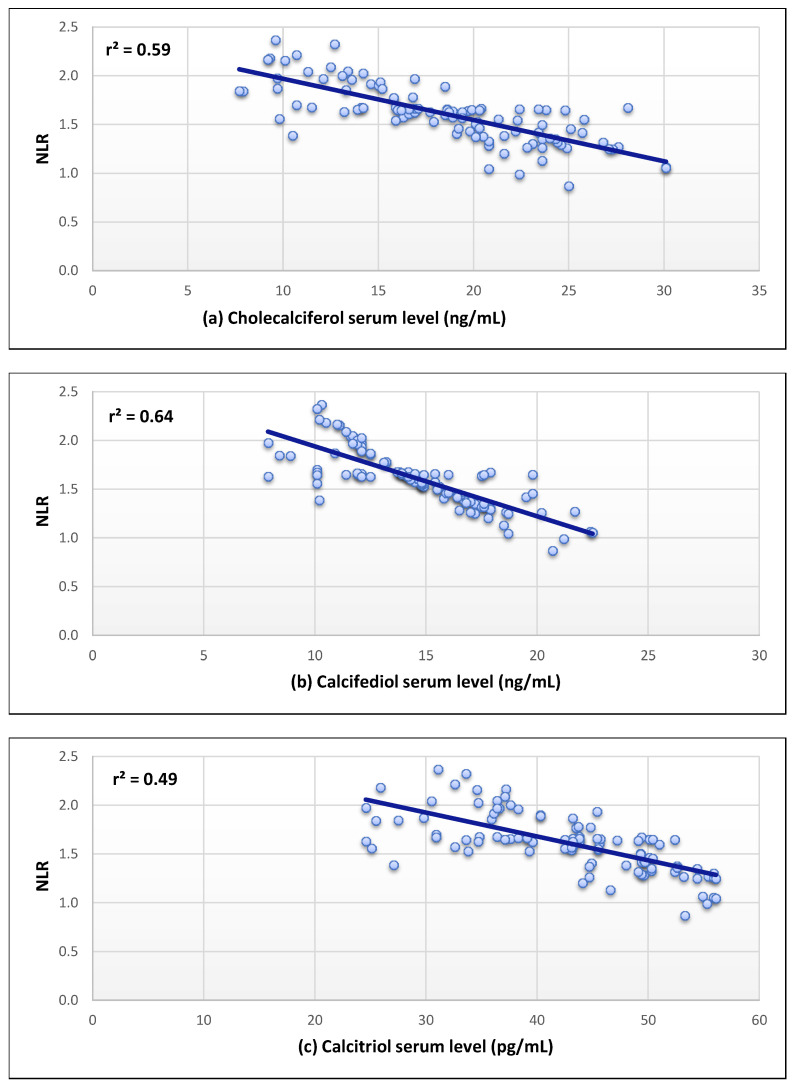
Correlation between the neutrophil-to-lymphocyte ratio (NLR) and (**a**–**c**) in COVID-19 patients.

The serum levels of the three forms of vitamin D and serum ACE2 levels were shown to have a substantial positive correlation (r^2^ = 0.63–0.77) as revealed in Figure 2. In addition, a strong negative correlation was found for the serum level of Il-6 (r^2^ = 0.75–0.83) (Figure 3). Moreover, a moderate inverse correlation was found between the vitamin forms and the NLR (r^2^ = 0.49–0.64), as seen in Figure 4.

## 4. Discussion

This study revealed a correlation between vitamin D deficiency, the severity of COVID-19, and specific inflammatory markers in COVID-19 patients. This relationship is based on the fact that vitamin D receptors are widely expressed in the human body and that active vitamin D compounds may have considerable biological effects on a variety of cellular signaling pathways [41]. Several studies have reported a correlation between patients’ history of vitamin D insufficiency and an increased chance of positive COVID-19 test findings [25,26,27,28]. The current research suggests that the low level of vitamin D3 and its metabolites is an indicator of worse COVID-19 illness consequences. The low levels of serum vitamin D metabolites detected during the acute COVID-19 infection, on the other hand, may be a result of persistent inflammation rather than an underlying cause [42].

As reflected by our results, it was noticed that the total WBCs, neutrophils, platelets, MCH, and MCHC were significantly elevated in COVID-19 patients in comparison with healthy subjects, which is consistent with earlier research [43]. This might be related to the virus’s effect on RBC and WBC development or degradation, or the presence of comorbidities. In contrast, lymphocytes, RBCs, and hematocrit were significantly lower in the patients group. The RBCs of COVID-19 patients were reported to have lower amounts of key antioxidant enzymes and higher levels of protein breakdown indicators [44]. Lymphocytopenia and thrombocytopenia were also increasingly found when comparing severe COVID-19 cases with non-severe ones [43].

In addition to a large drop in lymphocyte count, the patients group was noticed to have a considerable rise in neutrophil count. We found that NLR increased in COVID-19 individuals in comparison with the control individuals, with considerably higher levels in patients who died from the disease. The NLR was established as a valuable predictor of systemic inflammation and has been studied as a prognostic indicator for a variety of disorders such as sepsis and cancer [25]. In addition, the association between ARDS and elevated NLR has also been observed [25]. In the current study, moderate inverse correlations were found between serum levels of cholecalciferol, calcifediol, and calcitriol and the NLR. This could be due to type I interferon-dependent transitory lymphopenia, which is seen in a variety of viral infections. The direct viral infection via spike receptors in T lymphocytes may also contributes to lymphopenia [45]. These data may aid in predicting clinical severity in COVID-19 patients.

Our results also showed that serum ACE2 concentrations were significantly increased in the patients compared with the control individuals. Furthermore, they were significantly higher in patients with increased levels of cholecalciferol and its metabolites compared with the other COVID-19 patients. It is reported that increased ACE concentrations lead to increased levels of angiotensin II, which might be responsible for the severe lung injury in COVID-19 patients, while inhibiting the angiotensin II-signaling pathway and/or RAAS, through ACE2, has been shown to protect against lung damage [46]. The strong positive correlation (r^2^ = 0.63–0.77) between the ACE2 serum levels and levels of cholecalciferol and its metabolites in our COVID-19 individuals confirms the expected role of ACE level in the disease severity. These findings support the belief that vitamin D signaling may cause RAAS to respond and limit vitamin D’s function as a transcription factor in renin gene suppression, hence serving as a negative endocrine regulator on RAAS [47].

Additionally, the levels of IL-6 were observed to be significantly elevated in the COVID-19 patient group compared with the control group. The values were also higher in patients with vitamin D insufficiency. A strong negative correlation (r = 0.75–0.83) was found between cholecalciferol, calcifediol, and calcitriol and IL-6 levels. This can be explained by the significant role of vitamin D3 in reducing immune cell IL-6 synthesis, potentially lowering its pro-inflammatory effects without directly targeting IL-6 receptors, and preventing any negative effect on the IL-6 anti-inflammatory properties [34]. Vitamin D has also been proven to diminish TNF-α levels, implying another treatment approach for combating the COVID-19 cytokine storm [35].

This study showed that vitamin D3 and its metabolites were highly significantly lower in the patients group compared with the healthy controls. Vitamin D status has traditionally been determined using levels of calcifediol or, sometimes, calcitriol. The physiological sophistication of synthesis route of these metabolites implies that, in addition to measuring serum levels of one metabolite, efficient management of vitamin D hydroxylation may be significant [48]. Calcitriol and calcifediol, on the other hand, have distinct biological features and their concentrations are very loosely associated. Determining their ratio to each other is more effective than evaluating their levels separately. This may help estimate how well the vitamin D system is working to keep the picomolar levels of the active metabolite, calcitriol, stable, which is necessary for the predicted biological effects [24]. Two vitamin D metabolite ratios (VMR) were suggested to be suggestive of the effectiveness of vitamin D hydroxylation. VMR1 (calcitriol/calcifediol) theoretically depicts how much calcitriol, in picograms, is produced for every nanogram of circulating calcifediol and can be indicative of vitamin D hydroxylation efficacy at the kidney. On the other hand, VMR2 (calcifediol/cholecalciferol) shows how many nanograms of calcifediol are produced per nanogram of circulating cholecalciferol and can indicate vitamin D hydroxylation effectiveness in the liver. In this study, however, both ratios were found to be within the normal suggested ranges in both patient and control groups. Although both VMRs were higher in the COVID-19 patient group compared with the control group, the differences were nonsignificant. This may indicate that the levels of vitamin D and its metabolites decrease in the same manner in COVID-19 patients without a noticeable effect on the hepatic and renal hydroxylation efficiency.

## 5. Limitations of the Study

Although this study has shown interesting potential clinical implications, our sample involved only patients from the first wave of COVID-19. In addition, the sample size was relatively small. Therefore, the results cannot be generalized unless further multicenter studies with larger sample sizes are conducted covering patients affected by subsequent waves of the pandemic.

## 6. Conclusions

This study showed strong correlations between the serum levels of vitamin D3 (cholecalciferol) and its metabolites, calcifediol and calcitriol, with disease severity in COVID-19 patients. Moreover, certain inflammatory markers (especially Il-6 and ACE2) and clinical hematological outcomes of COVID-19 disease were well correlated with the levels of the vitamin and its metabolites. However, the renal and hepatic hydroxylation of the vitamin, represented by VMR, were not significantly affected during the course of the infection. These data may help to clarify logical therapeutic decisions in various patient populations.

## Figures and Tables

**Table 1:** Demographic features of COVID-19 positive group compared with control group.

Demographic Features	COVID-19 Group (n = 103)	Control Group (n = 50)	*p*-Value
Age (years) ^a^	47.32 ± 12.44	49.26 ± 9.94	NS
Sex ^b^			
*Male*	80 (77.67%)	37 (74.0%)	-
*Female*	23 (22.33%)	13 (26.0%)	-
Weight (kg) ^a^	73.90 ± 12.30	72.12 ± 12.89	NS
Height (cm) ^a^	165.86 ± 9.40	165.06 ± 7.52	NS
BMI (kg/m^2^) ^a^	26.74 ± 3.38	26.47 ± 4.43	NS

BMI, body mass index; NS, non-significant. ^a^ Mean ± standard error of the mean. ^b^ Frequency and ratio.

**Table 2:** Laboratory test results for COVID-19 positive group compared with the control group.

Test	COVID-19 Group * (n = 103)	Control Group * (n = 50)	*p*-Value
ACE2 (U/L)	72.37 ± 18.67	32.36 ± 11.27	≤0.001 (HS)
IL-6 (pg/mL)	95.84 ± 25.23	2.76 ± 0.62	≤0.001 (HS)
Glucose (mg/dL)	188.42 ± 37.62	125.75 ± 25.49	≤0.001 (HS)
Total WBCs (×10^3^/μL)	7.86 ± 2.59	5.18 ± 0.76	≤0.05 (S)
Neutrophils (%)	57.22 ± 2.27	47.81 ± 6.17	≤0.001 (HS)
Eosinophils (%)	5.1 ± 0.16	5.08 ± 0.17	NS
Lymphocytes (%)	36.63 ± 6.14	45.02 ± 2.64	≤0.001 (HS)
Platelets (×10^3^/μL)	264.42 ± 72.30	215.10 ± 25.38	≤0.001 (HS)
NLR	1.61 ± 0.30	1.07 ± 0.16	≤0.001 (HS)
RBCs (×10^6^/µL)	4.54 ± 0.56	5.09 ± 0.64	≤0.05 (S)
Hb (g/dL)	11.98 ± 1.78	13.25 ± 0.9	≤0.05 (S)
HCT (%)	36.25 ± 5.44	40.92 ± 2.21	≤0.05 (S)
MCV (fL)	83.79 ± 5.34	81.35 ± 3.47	NS
MCH (pg)	30.40 ± 3.46	27.21 ± 2.82	≤0.05 (S)
MCHC (g/dL)	34.00 ± 1.31	30.12 ± 1.75	≤0.05 (S)

ACE, angiotensin converting enzyme; Hb, hemoglobin; HCT, haematocrit; HS, highly significant; IL-6, interleukin-6; MCH, mean corpuscular hemoglobin; MCHC, mean corpuscular hemoglobin concentration; MCV, mean corpuscular volume; NLR, neutrophil-to-lymphocyte ratio; NS, nonsignificant; RBCs, red blood cells; S, significant; WBCs, white blood cells. * Mean ± standard error of the mean.

**Table 3:** Serum levels of vitamin D3 and its metabolites in COVID-19 positive group compared with control group.

Vitamin D and Its Metabolites	COVID-19 Group * (n = 103)	Control Group * (n = 50)	*p*-Value
Cholecalciferol (ng/mL)	18.50 ± 5.36	29.13 ± 4.94	≤0.001 (HS)
Calcifediol (ng/mL)	14.60 ± 3.30	23.10 ± 3.02	≤0.001 (HS)
Calcitriol (pg/mL)	42.90 ± 8.44	65.15 ± 7.11	≤0.001 (HS)
Cacitriol/Calcifediol ratio (VMR1, pg/ng)	2.97 ± 0.30	2.86 ± 0.46	NS
Calcifediol/Cholecalciferol ratio (VMR2, ng/ng)	0.82 ± 0.14	0.81 ± 0.17	NS

HS, highly significant; NS, Non-significant; VMR, vitamin D metabolite ratio. * Mean ± standard error of the mean.
